# Human Knockout Carriers: Dead, Diseased, Healthy, or Improved?

**DOI:** 10.1016/j.molmed.2016.02.006

**Published:** 2016-04

**Authors:** Vagheesh M. Narasimhan, Yali Xue, Chris Tyler-Smith

**Affiliations:** 1 Wellcome Trust Sanger Institute, Hinxton, Cambridge CB10 1SA, UK

**Keywords:** loss-of-function variants, gene function, clinical interpretation

## Abstract

Whole-genome and whole-exome sequence data from large numbers of individuals reveal that we all carry many variants predicted to inactivate genes (knockouts). This discovery raises questions about the phenotypic consequences of these knockouts and potentially allows us to study human gene function through the investigation of homozygous loss-of-function carriers. Here, we discuss strategies, recent results, and future prospects for large-scale human knockout studies. We examine their relevance to studying gene function, population genetics, and importantly, the implications for accurate clinical interpretations.

## Trends

Genome and exome sequencing are revealing many candidate loss-of-function (knockout) variants in every human genome.

Sequencing consanguineous populations is the most efficient way of discovering additional knockouts.

The phenotypic consequences of apparent knockouts are difficult to predict accurately because of (i) imperfect variant calling, gene annotation, and prediction of the molecular consequences at the RNA and protein levels, and (ii) variation in the biological consequences of knocking out different genes.

Human knockouts provide opportunities to investigate gene function and essentiality, as well as to suggest and validate potential drug targets.

The clinical interpretation of knockouts is complicated by all the above factors, in addition to variable penetrance and a lack of suitable databases.

Consistent calling, annotation, and database standards for variants are presently needed.

## The Need to Understand Knockouts

*Early in 2017, parents bring their newborn baby into Dr Wuhabi's clinic. There is nothing obviously wrong, but the parents are worried. Dr Wuhabi has the baby's genome sequenced: there are no variants from the actionable list, but a homozygous knockout of little-studied gene is called. How should she advise the parents?*

**Figure 1:**
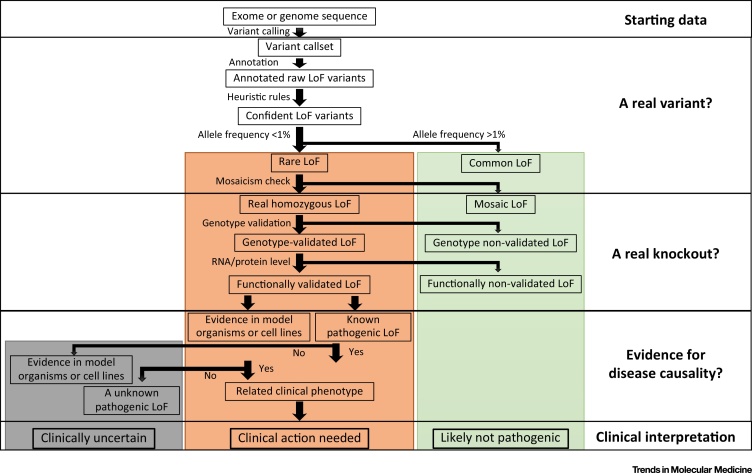
Key Figure: Steps for the Clinical Interpretation of a Genetic Variant Discovered in a Genomic Sequence of Interest

This scenario is imaginary, but may soon be reality. Knockouts of some genes in humans certainly cause genetic diseases, but for other genes the consequences depend on the genetic background or environment; yet other knockouts may have no detectable effect, or may even be beneficial. A flurry of recent papers has begun to reveal not only the prevalence of knockouts in the population, and their scientific interest, but also the complexity of understanding their medical implications. We review here these new developments, the steps necessary for their clinical interpretation (Figure 1, Key Figure), and consider possible future steps to resolve some of these complexities.

## Is It Really a Knockout?

**Figure 2:**
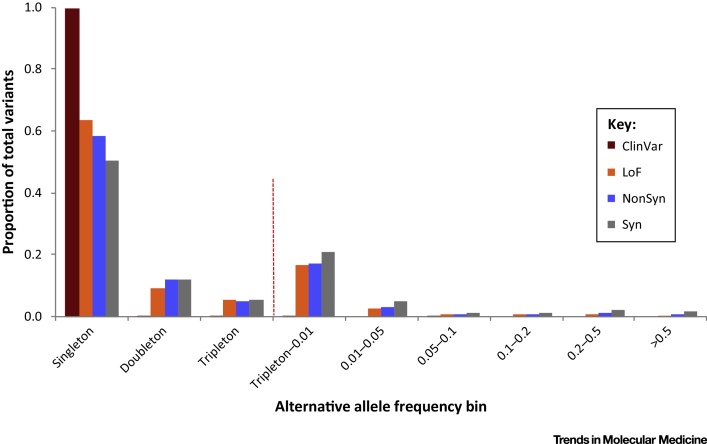
Allele Frequency Spectrum of Different Classes of Variants in the 1000 Genomes Project Data. Alleles were assigned to a bin according to their frequency in the study population, and the bins plotted in order of increasing frequency on the horizontal axis, with the functional classes being indicated by different colors within each bin. Singleton, doubleton, or tripleton variants refer to those seen only once, twice, or three times in the data, respectively. In this sample from apparently healthy populations, variants seen in disease databases such as ClinVar (ClinVar; dark red) are observed almost exclusively in single individuals. Loss-of-function variants (LoF; orange), which knock out genes and represent the most damaging functional class of variant, are also seen most often in only a single individual, although some are more frequent. Non-synonymous variants (NonSyn; blue), which change an amino acid in the protein, are on average present at higher frequency in the population, and are thus shifted towards the right-hand side of the plot. Synonymous variants (Syn; grey), which do not change an amino acid, have on average the highest allele frequencies.

While sequencing technology is becoming a ubiquitous part of genetic diagnosis, understanding the impact of the variation discovered on the human phenotype remains a challenge, as illustrated above. Naturally-occurring knockout or **loss-of-function** (LoF, see Glossary) variants (the terms are interchangeable), in other words genetic variants that are predicted to severely disrupt the function of human protein-coding genes [1], are often prime candidates for follow-up. However, significant difficulties remain: first, with the identification and **calling of DNA variants** and, second, with the **annotation** of whether they truly disrupt protein function or not (Figure 1). LoF variants as a class are rare (Figure 2) [2] and are poorly called by current methods. While **exome sequencing** and whole-genome sequencing technologies allow reliable calling of SNPs, calling small insertions and deletions remains a developing area. Moreover, differences in **coverage** as well as in an inability to span **breakpoints** decrease sensitivity for calling large structural variants [3]; these non-SNP variants make up a large fraction of naturally-occurring knockout variation and may still have high error rates. From a clinical perspective, validation of variants of interest (using an independent technology such as **Sanger sequencing** or **Sequenom genotyping**) is always needed, and must be part of standard practice [4].

Furthermore, when studying homozygous variants, the possibility of **mosaic** homozygous/heterozygous status due to **somatic crossover** needs to be considered. Similarly, compound heterozygous LoF variants in the same gene on different chromosomes knock out the gene, while equivalent variants on the same chromosome only knock out a single copy. Further, inconsistencies in gene reference sets and the annotation of protein-coding genes add an additional layer of complexity. There can be considerable differences [5] between knockouts that are called using different widely used gene models for human protein coding genes such as **RefSeq** [6] and **GENCODE** [7]. In addition, software packages used to derive the consequences of sequence variation on proteins, such as **Annovar** [8] or **Variant effect predictor** (VEP) [9], can produce further differences even when using the same gene models [10].

In addition to the subtleties in drawing up an initial list of knockout variants, predicting the effect of a specific variant on protein production and on the phenotypic consequences of an observed transcript reduction remain even more challenging. Transcript levels can readily be measured, and are relevant because large deletions may remove a transcript entirely, while smaller LoF variants may lead to nonsense-mediated decay (NMD) (Box 1) which reduces the transcript level. Surprisingly, even if genetic variation triggers NMD and there is degradation of the RNA, the reduction in RNA levels may not reduce the protein level [11]. Finally, the effect of alternative splicing may lead to partial LoF variants, which affect only a subset of the transcripts of a gene, meaning that a functional protein may still be produced from other transcripts. It is currently effectively impossible to assess the relative functional importance of different transcripts for most genes, and partial LoF variants can cause **Mendelian disease** [12]. To sidestep these limitations, strategies which filter variants based on deterministic rules that best predict true LoF behavior have been developed (LOFTEE: loss-of-function transcript effect estimator; https://github.com/konradjk/loftee) but their systematic evaluation using large-scale RNA and protein data is still incomplete. In settings where annotation is important for diagnosis, further confirmation of loss needs be obtained by direct observation of an absence of the protein product or activity from a suitable sample. Only then can we be fully confident of a knockout.

## Is it on the Disease-Causing List?

Many proteins are unnecessary for general life and good health: olfactory receptors, our largest gene family, provide a prime example [13]. Thus, even for a confirmed knockout, we still need to determine whether it has a relevant phenotypic effect. The traditional way to do this is to look in a list or database of known disease-causing variants. Decades of work by clinical geneticists and physicians have led to the compilation of such databases. The predominant approach has been to discover candidate causal genes/variants segregating in families and follow them by analyzing additional patients with similar phenotypes. After assessing the mode of inheritance (dominant, recessive, etc.), the presence of the same or equivalent variant (often LoF or a damaging amino acid substitution) in the same gene, and its absence from a sample of unaffected individuals, has been considered to establish causality. More recently, tools have been designed to enable computational prediction of mutations (Box 2).

Beyond simple Mendelian conditions, this approach has also been successful in identifying causal genes for more complex disorders by focusing on extreme and rare phenotypes. The first large-scale sequencing study performed in **consanguineous** families led to the identification of 50 novel candidate genes for developmental disorders [14]. This success was soon followed by the sequencing of an even larger cohort of 1113 **trios** and the implementation of a robust translational genomics workflow to allow feedback of potentially diagnostic findings to clinicians and research participants [15]. Importantly, by utilizing a genotype-driven approach to identify subsets of patients with similar disorders, the newly implicated genes increased by 10% the proportion of subjects who received a diagnosis [16]. As such, exome sequencing of single patients with extreme phenotypes has been applied more widely. For example, a knockout of the immune gene *IRF7* was shown to confer susceptibility to flu viruses, leading to life-threatening influenza in an otherwise healthy child [17].

In a similar vein, sequencing of fetuses lost preterm has identified novel knockout variants in *CHRNA1*, a muscle acetylcholine receptor, as a cause of lethal **fetal akinesia** [18]. More generally, family-based designs to uncover recessive forms of embryonic lethality by examining significant depletion of transmitted homozygote genotypes have implicated *THSD1*, a thrombospondin type 1 domain-containing protein of poorly understood function, as a candidate for a monogenic cause of embryonic lethality [19]. Taken together, Mendelian disease genes and embryonically lethal genes provide a spectrum of knockout variants ascertained as disease-causing by analyzing carriers of clinically diagnosed phenotypes. Further sequencing in this domain with larger sample sizes, better curation, and deeper phenotyping will steadily increase this catalog. Moreover, a complementary approach is to sequence healthy people: the knockouts they carry are unlikely to be disease-causing. However, that interpretation of such lists is not as simple as it seems.

## How Can We Best Discover More Knockouts?

The logical end to the approach described above is to discover knockouts in all of the 20 000 or so human protein-coding genes and classify them as either being lethal before birth, compatible with life but disease-causing, or as having no disease consequences. However, LoF variants typically have very low frequencies, meaning that very large sample sizes are required to systematically discover LoFs in every gene. With the cost of sequencing decreasing, there have been several approaches to uncover novel knockout variants on a large scale, using different strategies.

A simple approach is to collect a large number of individuals from multiple cohorts that have already been sequenced for diverse studies. The Exome Aggregation Consortium (ExAC) has put together such a collection of >60 000 exomes from a wide range of phenotypes and ages. This non-trivial exercise required performing reproducible variant calling and quality control across the entire set of exomes that have been sequenced on different platforms and time-periods [20]. At this scale, sequencing has been able to identify a variant in at least one individual at one in every eight bases of coding sequence, as well as many sites with recurrent mutations. This work has enabled us to understand the extent of **haploinsufficiency** in the genome with the observation that 3230 genes exist with a severe depletion of heterozygous knockout variants, most of which do not have an established human disease phenotype [21]. Given the large sample size of the data, it is also possible to investigate the tolerance to dominant consequences of knockouts of individual genes by employing a model that compares the synonymous mutational load with that of LoF mutations, taking into account gene length and base composition [22]. For example, an excess of LoF mutations in a particular novel gene for a disease cohort can indicate that certain mutations are disease-causing [23].

While sequencing individuals without selecting for particular population-genetic properties is an effective approach, such studies are in practice currently limited to the study of heterozygous LoF variants [24]. In randomly-mating populations, a variant present in 1 in 1000 individuals in a heterozygous state will only be present in 1 in 1 000 000 in a homozygous state, and discovering homozygous mutations by sequencing outbred individuals will therefore require very large sample sizes. Nevertheless, two complementary approaches have been used to discover rare homozygous knockouts.

**Bottlenecked** populations with extensive identity-by-descent (**identical-by-descent** genomic portions) present the most direct approach, and recently, ∼100 000 individuals from Iceland [25] and ∼30 000 individuals from Finland [26] (two such bottlenecked populations) have now been sequenced. Mildly pathogenic variants in small populations such as these are also more likely to drift to higher frequencies than in large populations, and association studies aiming to find pathogenic variation have also discovered knockout variants that lead to chronic disease. A striking example involved the identification of a LoF variant leading to insulin resistance, with an allele frequency of 17% in Greenland [27]. However, the potential of this strategy for discovering homozygous knockouts is limited by two factors. First, the portion of the genome that is identical-by-descent in these individuals, while higher than in outbred populations, is still small, especially when education programs reduce marriage between close relatives [28]. Therefore, the number of rare homozygous knockouts discovered per person is low. Second, the number of knockouts present in the entire population is limited to those present in founders (plus new mutations), and thus existing studies may already have discovered most of the LoF variation [26]. This would mean that future sequencing of individuals from these cohorts is less likely to yield novel mutations.

**Figure 3:**
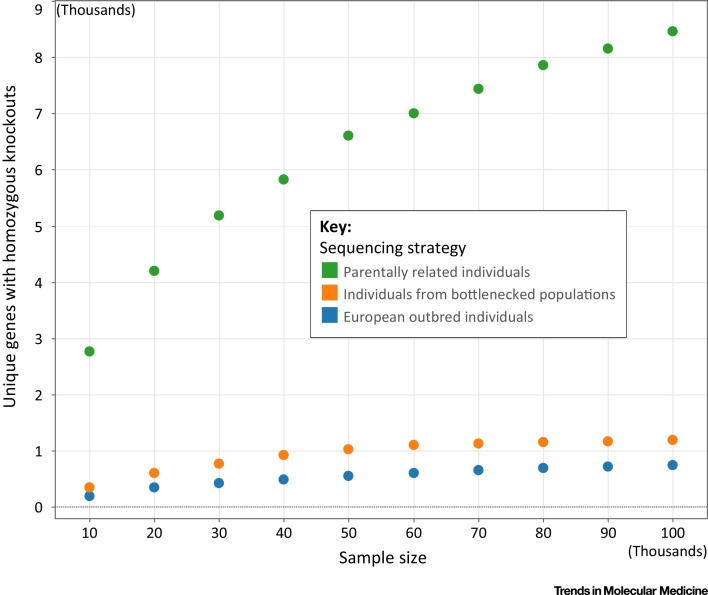
Number of Genes Carrying Homozygous Knockout Variants. The graph depicts such variants discovered by sampling populations with different structures and plotted as a function of sample size. Sequencing of parentally related individuals (green) provides discovery rates an order of magnitude higher than other strategies using outbred individuals (blue) or bottlenecked populations (orange). This implies that sequencing more parentally related individuals is the best future strategy.

An alternative approach is to investigate consanguineous populations, which have high degrees of parental relatedness, and large portions of their genome that are identical-by-descent because of family structure in the immediate preceding generations. Two recent studies have sequenced individuals of Pakistani descent and shown that one in every two individuals who are the offspring of first cousins has a rare knockout variant 29, 30. This rate is almost 50-fold higher than that discovered in bottlenecked populations. Reassuringly, overlap of genes from the datasets that have been produced using this approach suggests that rare LoF variants are often not shared between populations and that the rate of discovery of knockouts from consanguineous cohorts is sufficiently high to increase our understanding of homozygous knockouts substantially (Figure 3).

## How Can We Investigate the Phenotypic Consequences of Knockouts?

Although we have catalogs of knockout variants, and strategies for large-scale discovery of more such variants, understanding the impact of gene knockouts, and thereby gene function, is considerably more difficult. Large cohorts with linked health records evaluating gross patient phenotypic status have been examined in recent studies 24, 29. However, information on particular knockouts or genes remains difficult to extract because these knockouts are generally extremely rare and may be seen only in a single individual. Because the ascertainment is based on the genotype, recall and deep phenotyping are often required. Once a particular knockout is identified, family-based designs can potentially be used to ascertain more individuals sharing the same (heterozygous or homozygous) variant. An example of this strategy was demonstrated in the discovery of a rare complete knockout in *APOC3*, which encodes an LDL protein, where a single individual with extremely low fasting triglyceride levels from a remote village in Pakistan was initially identified [29]. His extended family was later contacted and four more homozygous knockout individuals from the large pedigree were found. This greatly improved the association signal and provided evidence implicating *APOC3* in the control of triglyceride levels in the blood [29]. Similarly, a homozygous knockout variant of *PRDM9* (PRDM9 directs and initiates recombination in mammalian cells) was found in one woman from a cohort of 3222 individuals [30]. Follow-up by **single-molecule phasing** of her genome, together with that of her child, validated the predicted altered recombination pattern, and thus revealed PRDM9 redundancy in humans [30]. These discoveries illustrate the effectiveness of deep phenotyping of individual gene knockouts discovered through population sequencing because these tie together patient, epidemiological, molecular, and electronic health record data in the identification of novel biological functions for human genes.

Alternatively, cellular assays or model organisms can be used to provide evidence of variant pathogenicity by showing that a knockout variant alters gene function with consequences that mimic a disease phenotype, and that these differences are rescued by methods that recover the wild-type function. This approach, together with the ability to generate knockout mutations rapidly, has allowed the testing of synthetic lethality in human cell lines. In the past year, this has been investigated at large using **CRISPR/Cas9** and whole-genome **gene-trap assays** to screen for genes required for proliferation and survival in near-haploid KBM7 chronic myelogenous leukemia cell lines 31, 32. These studies have highlighted approximately 2000 genes essential to human cellular function in these systems, which in fact parallel those found in yeast [33]. Such analyses have allowed us to further understand the phenotypic consequences of gene knockouts.

## How Should We Interpret Knockouts in the Clinic?

**Figure 4:**
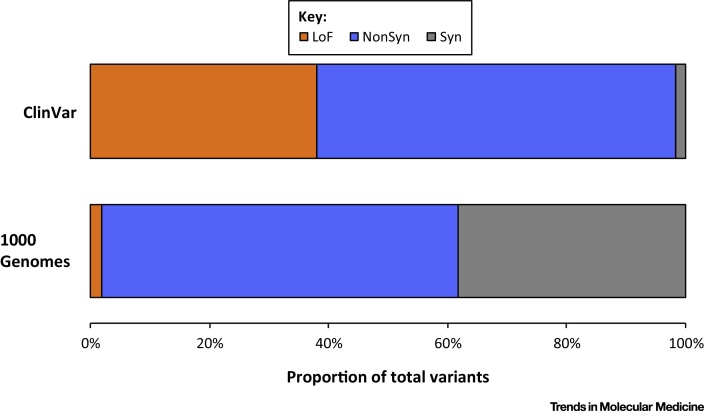
Proportions of Different Variant Classes in the General Population. The graph provides data from the 1000 Genomes Project, Phase 3 (lower bar), and the ClinVar database of disease-associated variants (ClinVar; upper bar). Non-synonymous variants (NonSyn; blue) are abundant in both samples; synonymous variants (Syn; grey) are abundant in the general population, but seldom cause disease; LoF variants are scarce in the general population but form a high proportion of ClinVar entries (LoF, orange). This shows that, although knockout variation is present at low frequency in the general population, it has a substantial impact on disease.

The biggest challenge, however, lies in how we interpret the effect(s) of a variant on health-related phenotypes because these are often moderated by other genetic variants or by the environment. This variability in the resulting phenotype, known as incomplete **penetrance** [34], makes the interpretation and **actionability of knockout variation** particularly challenging. Several online databases exist to annotate the clinical relevance of genes or variants and the effect of knockout variation on phenotype. The widely used databases Online Mendelian Inheritance in Man (OMIM; http://omim.org/), The Human Gene Mutation Database (HGMD) [35], and ClinVar [36] rely largely on cases reported in the literature, and LoF variants are major components of their lists (Figure 4); but, as discussed above, these are generally ascertained from affected individuals and their penetrance is often poorly understood. Moreover, some of the reported disease genes and variants may only include evidence from a single individual or family. However, sequencing-initiated population screens, which are mostly recruited from healthy cohorts, present a contrasting ascertainment by detecting the variant independently of its penetrance. Moreover, we are learning that incomplete penetrance may be the rule rather than the exception. For example, knockouts in *GJB2*, which encodes a gap junction subunit expressed in the developing cortex, and which cause hearing loss, have been widely studied and accepted as a clear Mendelian condition with high penetrance; however, population screens have revealed the existence of individuals harboring knockouts who exhibit normal audiometry [30]. Another example involves a knockout variant in *KMT2F*, a gene which forms part of a histone methyltransferase (HMT) complex that methylates histone H3 at Lys4. This same variant has been implicated in a large case–control schizophrenia study, as well as in probands with intellectual disability, thus making the diagnosis of the disease associated with the genotype difficult to determine [37]. Generally, when only phenotypic information about a few individuals with a particular genotype is available, and the phenotypes differ, predicting phenotype from genotype may be virtually impossible.

In light of these complexities, there is great need for consolidated approaches to sharing information in a reproducible manner. Consolidated data should include information ranging from read information and quality metrics of the sequence data to knockout allele frequencies in different cohorts and health status of the carrier individuals. Crucially, as recent reviews on clinical actionability suggest 38, 39, 40, 41, there is a need for scoring LoF variants, including those of the same gene, on a quantitative scale from benign to pathogenic. It is essential for the information to be curated in such a manner that crucial data, both in terms of observational phenotypes as well as quantitative measurements, are aggregated into a framework [42]. The scoring schema should reflect study design, gene and variant level data, publications and databases, as well as clinical diagnosis. This would allow translation of genomic research findings into the clinical diagnostic setting and empower informed decisions about actionability [42].

## What Can We Learn about the Population Genetics of Knockouts?

Outside the medical domain, there is great interest in understanding the extent and impact of LoF variants from a population-genetic perspective. The average number of LoFs per person (∼100) in populations from Africa, Europe, and East Asia, and their characteristics of low allele frequency and type (less than half of LoF variants are SNPs), were discovered by sequencing the first 150 individuals in the 1000 Genomes Project [43].

Further sequencing in control cohorts has provided a better understanding of the portion of the genome that is essential [44], both in terms of genes that are haploinsufficient as well as those that are recessive. By examining the effects of purifying selection (Box 3), which removes strongly deleterious LoF variants, we can identify a set of genes under evolutionary constraint. These genes are also more likely to contribute to human disease [45]. We have also been able to measure the effect of purifying selection directly; there is now a better estimate of lethal equivalents or, rather, of the human mutational load of heterozygous mutations that would be lethal if homozygous, from looking at (i) severe disease cases in founder populations [46], or (ii) consanguineous pedigrees with a deficit in homozygous genotypes [30]. These studies have determined that any human individual carries, on average, between one and two recessive lethal variant equivalents per genome.

## How Are Knockouts Useful?

Perhaps the study of gene knockouts is most useful when examining instances where a naturally-occurring LoF variant proves beneficial to health. Notable examples include lowering LDL levels (*PCSK9*), decreasing susceptibility to HIV (*CCR5*), increasing endurance (*ACTN3*) and increasing sepsis resistance (*CASP12*) 47, 48, 49. These discoveries have not only stimulated drug development but have also prompted further genetic testing of these genes; for instance, additional modifying alleles of *CCR5*, linked to HIV susceptibility, were identified in African populations [50].

Drug safety checks are a crucial component of the clinical trial process, and the majority of compounds that enter trials fail to demonstrate safe use and are then abandoned, often after considerable expense. Naturally-occurring variants in humans affecting the activity or dosage of a particular gene or protein can be used in effective drug screens before embarking on clinical trials, serving in the determination of drug toxicity parameters [51]. This approach is exemplified by lipid genes, where longstanding cohort studies have shown the benefits of lowering cholesterol levels. For example, in addition to the *PCSK9* knockouts mentioned above, *APOC3* knockouts have been assessed – APOC3 deficiency has been shown to lead to reduced triglyceride levels in humans [52]. In both cases, humans with knockouts live long healthy lives, strongly suggesting that drug-mediated reductions in protein levels should be safe [53]. Importantly, genetics can also inform drug efficacy when the phenotype of heterozygous and homozygous knockouts can mimic dose–response curves. For example, the drug darapalib, aimed at treating atherosclerosis [54], failed to pass drug trials, exemplifying a case where large-scale clinical trials across tens of thousands of people could have been avoided if only the genetic screen showing a lack of molecular phenotype could have been first examined.

Another important use of knockouts involves the identification of modifier genes via variation in penetrance. In one application of this principle, the genomes of individuals carrying knockouts without the expected disease phenotype can be searched for naturally-occurring compensatory or modifying variants. Such studies have, for example, revealed secondary variants in fetal globin genes that modify the severity of sickle cell disease by ameliorating the effect of the primary causal variant in the β-globin gene [55]. A study studying symptom-free adults is now under way to systematically search for such ‘resilience’ variants modifying early-onset childhood disorders in a set of diseases known to have a single monogenic cause 56, 57.

## Concluding Remarks

Research on human gene knockouts, as well as on their phenotypic and clinical interpretation, is very active. It is leading to the identification of an increasing number of variants and, consequently, the need for eliciting clinical action or not is becoming clear, even if many questions remain in the field (see Outstanding Questions). Noteworthy is the fact that, with a population size of seven billion people worldwide, multiple knockouts of every human gene will have arisen from new mutations in the last generation of conceptions. Fortunately, we now have the technologies to continue analyzing and understanding such genetic mutations.

*After checking the validation data for the gene knocked out in the baby, Dr Wuhabi looks it up in the new online OKOD (Online KnockOut Database). There are two entries: an English woman aged 55 years homozygous for a premature stop codon recorded as having two children, with medical details ‘to be added’, and a Chinese man aged 92 years heterozygous for a deletion and a splice-site variant in separate copies of the gene, recorded only with age-related hearing loss. Dr Wuhabi reassures the parents that knockout of this gene is associated with normal life, and that the genome sequence gives her no cause for concern*.

This imaginary scenario is less plausible than our introductory one. Nevertheless, an increasing community of patients, healthy volunteers, medical and scientific professionals, as well as funders, could make this happen.

Outstanding Questions When does a candidate knockout variant identified in a DNA sequence result in absence of the protein product?

When and how does the full knockout of a protein product influence the phenotype of the carrier?

How many human gene knockouts are (i) lethal before birth, and thus are never observed; (ii) invariably or usually disease-causing; (iii) neutral, with only subtle effects on the phenotype; or (iv) beneficial to the carrier?

How much do the consequences (i–iv) vary between individuals, and how does this depend on the genotype background, environment, or other factors?

What are the best ways to standardize knockout identification, annotation, and database structure to support accurate clinical interpretations?

Could general drug-based approaches to reversing knockouts (e.g., readthrough of premature stop codons) be effective?

## Box 1 Understanding Nonsense-Mediated Decay (NMD)

The NMD pathway is found in all eukaryotes; its main function is to degrade and eliminate mRNA molecules that contain aberrant stop codons. This protects against the production of aberrant proteins which may be harmful. Despite years of effort and the formulation of rules to predict when NMD will be triggered, predictions remain unreliable [59]. The recent discovery of a protein that prevents mRNA degradation, PTBP1, is therefore of considerable interest. It has been reported that, when bound near a stop codon, PTBP1 blocks the NMD protein UPF1 from binding to 3′-untranslated regions (UTRs). PTBP1 can thus mark natural stop codons and prevent their degradation, allowing NMD to act on transcripts with premature stop codons and thus degrade aberrant mRNAs [60].

## Box 2 Computational Prediction of Phenotypic Consequence of Variation

Recently, several tools have been designed to predict phenotypic consequence for knockout variation, for example the genome-wide annotation of variants (GWAVA) score, and the Combined Annotation-Dependent Depletion (CADD) score 61, 62, allowing novel variants to be assessed. These tools are primarily variant-driven and determine pathogenicity by looking at sequence context, evolutionary constraint, and their impact on proteins. However, *in vivo* studies in model organisms have shown that these methods have high false-positive rates [63]. Complementary methods that utilize gene-level methods such as **residual variation intolerance score** (RVIS [64] and **gene damage index** (GDI) [65] have also been used for this purpose, and recent work has investigated the utility of gene-level thresholds in improving predictivity [66]. Furthermore, individual genes can be assessed together with others in close biological proximity to refine their phenotypic effect, as well as their susceptibility to disease, with network-based approaches such as the Human Gene Connectome Map [67].

## Box 3 The Impact of Demography on the Efficacy of Selection

In the past few years there has been much debate about the impact of demography on the efficacy of selection because allele frequency changes as a result of random genetic drift are expected to be greater in small populations, and thus selection less effective. In human populations, genetic effective population sizes within Africa are generally larger than outside, and so demographic impact on selection has therefore been evaluated by comparing populations within and outside Africa 68, 69, 70, 71. The ability to detect selection in genetic sequence data depends on the selective coefficient (measuring how strong selection has been), the mode of action of the variant (dominant or recessive), and the time over which selection has occurred. Inbred populations such as those sequenced to ascertain homozygous knockout variation represent a significant deviation from the demography of other human populations and can be used to measure this effect. In this setting, substantial portions of the genome of each individual may be identical-by-descent, and thus all variants in the population are often present in a homozygous state. Severely disadvantageous LoF variants, even if recessive, therefore manifest their phenotype, and are removed by natural selection, perhaps even in a single generation. This ‘purging through inbreeding’ leads to a lower number of LoF variants per individual than in an equivalent non-inbred population [30]. While observed in closely related species, most strikingly in mountain gorillas which have had extreme levels of inbreeding over long timescales [72], empirically observing this purging in humans has been difficult, although is expected to occur.

## Glossary

Actionability of knockout variation: the ability to use genotype data to change clinical management or therapy.
Annotation: the description of genes and other elements in the genome as well as their functions, including the likely functional impact of variants [58].
Annovar: a tool to functionally annotate genetic variants detected in diverse genomes.
Bottleneck: a severe reduction in size of a population, often short-term and followed by an expansion.
Breakpoint: the location at which a recombination event occurs between two genomic locations or chromosomes.
Calling of DNA variants: identifying the nucleotide or structural differences between a sequence of interest and the reference sequence.
Consanguineous: a pedigree in which the sampled individual has parents sharing a recent common ancestor.
Coverage: the number of sequence reads covering a particular position in the genome.
CRISPR/Cas9: bacterial clustered regularly interspaced short palindromic repeats (CRISPR) used with the Cas9 (CRISPR-associated) enzyme for efficiently editing genetic material.
Exome sequencing: technique for enriching and sequencing most or all of the protein-coding gene segments (exons) in a genome.
Fetal akinesia: term used to describe a clinically and genetically heterogeneous constellation of conditions that exhibit growth retardation and developmental anomalies.
Gene damage index (GDI): a process to score human genes based on their accumulated mutational damage, as assayed on the variation from the 1000 Genomes Project and their CADD scores (combined annotated dependent depletion), measuring deleteriousness of single-nucleotide or insertion/deletion variants.
GENCODE: set of high-quality gene reference annotations and their experimental validation for human and mouse genomes.
Gene-trap assay: a high-throughput approach to introduce insertional mutations into a mammalian genome.
Haploinsufficiency: the state in a diploid organism where a single functional copy of a gene (with the other copy inactivated by mutation) does not produce enough of its product (typically a protein) to lead to the wild-type condition, generating an abnormal or diseased state.
Identical-by-descent: portions of the genome where the maternal and paternal copies have identical sequences owing to inheritance from the same common ancestor.
Loss-of-function (LoF): variants causing the reduction or complete loss of a gene product, thereby impairing its biochemical function. Note that LoF variants are often only predicted LoF variants.
Mendelian disease: a genetic disease determined by a single locus, exhibiting an inheritance pattern that follows the laws of Mendel.
Mosaic: the presence of two or more populations of cells with different genotypes in one individual.
Penetrance: the chance that a genotype results in particular phenotype.
RefSeq: an annotated and curated collection of publicly-available nucleotide sequences (DNA, RNA) and their protein products.
Residual variation intolerance score (RVIS): a gene-based score intended to rank genes in terms of whether they have more or less common functional genetic variation relative to the genome wide expectation given the amount of apparently neutral variation the gene has.
Sanger sequencing: a method of DNA sequencing based on the selective incorporation of chain-terminating dideoxynucleotides by DNA polymerase during *in vitro* DNA replication, established by Fred Sanger and often used for small-scale genotype validation.
Sequenom genotyping: a method of genotyping by extending oligonucleotides with the single nucleotide of interest followed by determining the mass (and hence nucleotide added) by mass spectrometry, often used for medium-scale genotype validation.
Single-molecule phasing: a method for sequencing individual long molecules of DNA and thus identifying the set of variants that a single molecule (and thus single chromosome) carries (the phase of these variants).
Somatic crossover: genetic recombination (crossover) in somatic cells (the soma), contrasted with recombination during meiosis in germ cells.
Trios: three individuals, consisting of a mother, a father, and their child.
Variant effect predictor (VEP): a tool within ENSEMBL for the functional annotation of variants.
